# Methods for the evaluation of the Jamie Oliver Ministry of Food program, Australia

**DOI:** 10.1186/1471-2458-13-411

**Published:** 2013-04-30

**Authors:** Anna Flego, Jessica Herbert, Lisa Gibbs, Boyd Swinburn, Catherine Keating, Elizabeth Waters, Marj Moodie

**Affiliations:** 1 Deakin Health Economics, Faculty of Health, Deakin University, Melbourne, Australia; 2 Jack Brockhoff Child Health and Wellbeing Program, The McCaughey Centre, Melbourne School of Population Health, The University of Melbourne, Melbourne, Australia; 3 WHO Collaborating Centre for Obesity Prevention, Faculty of Health, Deakin University, Melbourne, Australia; 4 School of Population Health, Faculty of Medical and Health Sciences, University of Auckland, Auckland, New Zealand

## Abstract

**Background:**

Community-based programs aimed at improving cooking skills, cooking confidence and individual eating behaviours have grown in number over the past two decades. Whilst some evidence exists to support their effectiveness, only small behavioural changes have been reported and limitations in study design may have impacted on results.

This paper describes the first evaluation of the Jamie Oliver Ministry of Food Program (JMoF) Australia, in Ipswich, Queensland. JMoF Australia is a community-based cooking skills program open to the general public consisting of 1.5 hour classes weekly over a 10 week period, based on the program of the same name originating in the United Kingdom.

**Methods/Design:**

A mixed methods study design is proposed. Given the programmatic implementation of JMoF in Ipswich, the quantitative study is a non-randomised, pre-post design comparing participants undergoing the program with a wait-list control group. There will be two primary outcome measures: (i) change in cooking confidence (self-efficacy) and (ii) change in self-reported mean vegetable intake (serves per day). Secondary outcome measures will include change in individual cooking and eating behaviours and psycho-social measures such as social connectedness and self-esteem. Repeated measures will be collected at baseline, program completion (10 weeks) and 6 months follow up from program completion. A sample of 250 participants per group will be recruited for the evaluation to detect a mean change of 0.5 serves a day of vegetables at 80% power (0.5% significance level). Data analysis will assess the magnitude of change of these variables both within and between groups and use sub group analysis to explore the relationships between socio-demographic characteristics and outcomes.

The qualitative study will be a longitudinal design consisting of semi-structured interviews with approximately 10-15 participants conducted at successive time points. An inductive thematic analysis will be conducted to explore social, attitudinal and behavioural changes experienced by program participants.

**Discussion:**

This evaluation will contribute to the evidence of whether cooking programs work in terms of improving health and wellbeing and the underlying mechanisms which may lead to positive behaviour change.

**Trial registration:**

Australian and New Zealand Trial registration number: ACTRN12611001209987.

## Background

Cooking skills programs have been described as a practical illustration of how to simultaneously change knowledge, attitudes and behaviours around healthy eating practices [1]. Interest in cooking has been stimulated by media attention afforded to celebrity chefs and popular prime time television cooking programs. However, the need to promote cooking skills to individuals has in part stemmed from a decline in the traditional pathways by which individuals learn to cook [2], and from the hypothesis that a decline in cooking skills may have contributed to the growth in nutrition-related disease in certain sub-sections of western populations [3]. In Australia, Winkler investigated the relationship between a lack of confidence to cook and the purchasing of fruit and vegetables. The author concluded that cooking skills may contribute to socio economic differences in dietary intake and that promotion of such could be a useful strategy to improve fruit and vegetable intake [4].

In the past two decades, there has been an increase in the number of not-for-profit community-based cooking skills programs both in Australia and internationally [5-8]. Such programs have been conducted in a variety of community and institutional settings, targeting different sub-populations and varying in purpose; however, they are predominantly aimed at increasing confidence to cook, promoting healthy eating, addressing health inequalities and increasing access to healthy food [9].

Whilst there is emerging evidence of the effectiveness of these adult programs in terms of increasing confidence to cook and creating positive dietary change, this evidence, to date, has been based on small scale evaluations that are subject to methodological limitations [9]. In a recent systematic review of the effectiveness of adult community cooking programs conducted in the United Kingdom, only one evaluation was identified as suitably robust to provide reliable findings with respect to program effectiveness [10]. This highlights the need for more rigorous, larger scale studies to examine the range of impacts and outcomes of cooking skills programs and the underlying potential mechanisms for change in individual behaviour. At the same time, study designs must take account of the challenges associated with evaluation in community settings and be practical, feasible and sensitive to all stakeholders involved.

### The Jamie Oliver Ministry of Food program, Australia

This methods paper describes the evaluation framework and design for the Jamie Oliver Ministry of Food program (JMoF) Australia, Ipswich site. The JMoF program was originally developed by Jamie Oliver, a renowned celebrity chef and food author based in the United Kingdom (UK). JMoF Australia has been specifically adapted for the Australian setting. It is a community focused program that teaches basic cooking skills and good nutrition to non-cooks. It consists of 10, weekly, 1.5 hour cooking skills classes aimed at getting people of all ages and backgrounds cooking simple, fresh, healthy food quickly and easily [11]. Participants pay AUD10 per class and, where this may pose a barrier to entry, subsidies are made available.

JMoF was pioneered as a community-based cooking skills program in Rotherham, UK in 2008 and since then, other centres have opened in Bradford and Leeds and a mobile centre in the North West of the UK. These centres were reliant on funding mostly from local councils and to a lesser extent, charities and the private sector. The first Australian site opened in Ipswich in the state of Queensland in April 2011 co-funded by a local philanthropic non-government organisation (NGO), The Good Foundation (TGF), and the Queensland Department of Health. Ipswich was intentionally chosen given its significant low socio-economic status population [12] and increasing levels of overweight and obesity [13].

### Objectives of the JMoF program Australia

Consultation occurred between TGF, Queensland Health (as program co-founder) and the program evaluation team to describe program objectives of the JMoF program Australia in sufficient detail to be tested in an applied evaluation. The following program objectives resulted:

1. To provide opportunities, to people of different age and demographic background, to experience and learn how to cook healthy meals quickly and cheaply.

2. To increase program participants’ cooking skills, knowledge and self-efficacy.

3. To increase program participants’ enjoyment of food and social connectedness.

### Theoretical perspectives

**Figure 1:**
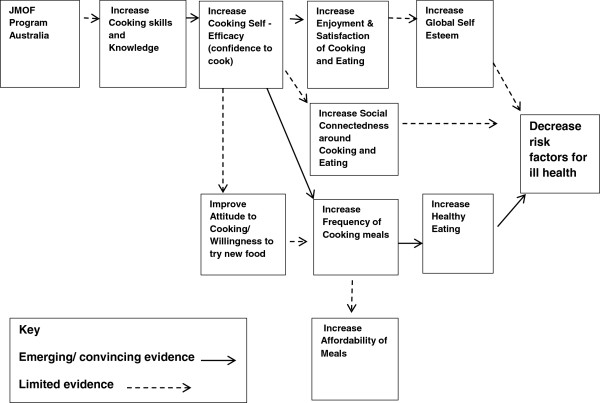
JMOF Australia program logic model.

A program logic model was developed as a framework to describe the potential pathways to behaviour change, and in turn to guide evaluative enquiry (Figure 1). Whilst some steps along the logic pathway were grounded by emerging or convincing evidence, other areas were backed by limited evidence, thereby requiring further hypothesis testing.

Theoretical frameworks were not explicitly stated for the JMoF Program. However, Carahar and Lang, 1999 [2] have identified theoretical perspectives specific to cooking skills that are in keeping with the objectives of the JMoF program and its evaluation - cooking skills empower individuals in preparation for healthy eating, encourage self-esteem and provide opportunities for leisure and enjoyment.

Other theories that resonate with the program include Kolb’s concept of experiential learning [14] which identifies the importance of empowering participants with practical “get your hands dirty” experience in learning to cook from scratch as a basis for skill acquisition, and Bandura’s Social Cognitive Theory [15] which states that changes in attitudes and beliefs and the development of self- efficacy (i.e. confidence in cooking) are central to influencing behaviour change. Bandura’s Social Learning Theory [16] also states that modelling is an important component of the learning process and that opportunities for practising learned behaviours and positive reinforcement are needed for learning to take place. An important element in the learning process is role model credibility [17].

## Methods/Design

The evaluation will be conducted over a 2.5 year period from late 2011 to early 2014. The evaluation was approved by the Deakin University Human Research Ethics Committee (HEAG-H 117_11) in October 2011. Evaluation project governance will be provided by a reference group (comprising personnel from the TGF team and the research team members) which will meet twice yearly to oversee the project. A representative from Queensland Health will be invited to attend Reference Group meetings, when appropriate.

The evaluation will use a questions-oriented approach [18] derived primarily from the JMoF Program objectives. It will also incorporate additional economic questions of relevance to potential government funders and program stakeholders. A longitudinal mixed methods evaluation design will be employed. The quantitative and qualitative components will be conducted sequentially, with baseline quantitative data informing sampling for the initial qualitative interviews. Each component will then be analysed independently, with merging of data occurring at the interpretation stage [19].

### Quantitative study

### Research questions

The quantitative component of the evaluation will answer the following research questions:

1. Does the JMoF program increase participants’ skills, knowledge, attitudes, enjoyment and satisfaction of cooking and cooking confidence (self-efficacy)?

2. Does the JMoF program result in broader positive outcomes for participants in terms of behaviour change to a healthier diet, more affordable healthy meals, improved self-esteem and social connectedness?

### Outcome measures

There will be two primary outcome measures: a change in cooking confidence (self-efficacy) and a change in self-reported mean vegetable intake (serves per day). Secondary outcome measures will include change in self-reported measures of: (i) mean daily fruit intake, (ii) mean weekly takeaway/fast food intake, (iii) frequency of cooking the main meal from basic ingredients, (iv) nutrition knowledge, (v) attitudes towards cooking, (vi) willingness to try new foods and (vii) enjoyment and satisfaction of cooking. Change in psycho-social measures such as (viii) global self-esteem and (ix) social connectedness in relation to cooking and eating will be measured as will (x) a change in participant’s total expenditure on food.

### Study design

A quasi-experimental pre-post design will consist of an intervention group of participants undergoing the JMoF program and a control group comprising of participants from the program waitlist who are waiting for at least 10 weeks until program entry. Recruitment to each group will be based on program start date and will not be subject to randomisation. Randomisation was not possible as it would not allow participants any choice as to when and with whom they participated in the program – which are important aspects of the JMoF program design [20].

Intervention participants will be surveyed at three time-points: program commencement, program completion (10 weeks) and at six months post program completion. Controls drawn from the waitlist will be surveyed at two time-points: 10 weeks prior to program commencement and on completion of their 10 weeks on the waitlist (which will correspond to their program entry). A time-three measurement will not be obtained from controls as it was considered neither feasible nor acceptable for the waitlist controls to have to wait a further six months before entering the program (equivalent to the intervention follow-up period); this potentially would lead to a high drop-out rate both from the evaluation and the program itself. However, for one of the primary outcome measures, vegetable intake, Queensland state-wide monitoring data will be used as a proxy time-three measure for the control group.

### Survey instrument

In collaboration with key stakeholders, a quantitative measurement tool was developed. The self- administered questionnaire was designed to be completed in approximately 15 minutes. Given the lack of validated and reliable survey tools which can accurately measure the impacts and outcomes of cooking skills programs in varying population groups [21], a prototype questionnaire was designed to address the unique objectives of the evaluation. Where suitable, specific questions were incorporated that have been previously used to measure the impact of cooking skills programs, particularly on cooking confidence and cooking behaviours such as those used by Barton et al, 2011 [21].

To measure the primary outcome of cooking confidence as a proxy for cooking self-efficacy, questions were developed addressing confidence in relation to specific cooking skills based on Short’s work [22] and Barton et al. 2011 work [21]. These items are presented on a 5 point Likert confidence scale ranging from ‘not at all confident’ to ‘extremely confident’.

The other primary outcome measure of change in vegetable intake will be captured through self-report questions of vegetable intake (serves per day) and aligned with baseline measurement questions of the same nature used by Queensland Health in its population-based self-reported health surveys [23]. Survey items addressing specific secondary outcome measures such as self- reported mean daily fruit intake and mean daily takeaway/fast food intake were also aligned with corresponding questions from the same baseline population health survey [23].

Other secondary outcome items include measuring change in the self-reported frequency of cooking the main meal from basic ingredients and the inclusion of salad or vegetables with the main meal. Nutrition knowledge questions, aligned with the nutrition messages embedded within the program, will test knowledge around salt, fat and sugar intake and have been adapted from Parmenter et al’s work [24].

Participant attitudes towards cooking and eating healthy foods, willingness to try new foods, enjoyment and satisfaction in cooking and eating healthy foods will be tested, using Likert scale based questions. Questions about shared enjoyment of cooking, eating and normative eating behaviours were adapted from questions from The Stephanie Alexander Kitchen Garden Evaluation (SAKG) [25]. The Rosenberg Self-Esteem Scale (RES), a widely validated and reliable measure of self-esteem, will also be administered [26]

Participants will be asked to report their total household food and drink (excluding alcohol) expenditure and expenditure specifically on take-away food and fruit and vegetables. Height and weight will be self-reported to enable the calculation of Body Mass Index (BMI).

### Piloting

The questionnaire was piloted in a 3 step process to test the design, content and potential delivery methods. Following comment by the reference group and academic colleagues on content and layout/design, a paper-based version of the questionnaire was piloted with a sample (N = 30) of the current JMoF population. Feedback was invited through informal focus group sessions, facilitating the identification of any questions that were ambiguous, sensitive or required further development.

The final stage of piloting involved testing the questionnaire in an online format whilst simultaneously testing the online survey distribution system and the likely response rate. As the survey distribution required integration between the JMoF participant database and the survey platform, Qualtrics™ [27], the piloting tested that these two components were effectively integrated and capable of providing the necessary information needs.

### Recruitment

All participants registered on the JMoF Australia waitlist database, aged 18 years or over, and who had received a confirmed start date for the program, will be eligible to participate in the quantitative component of the evaluation. Whilst the program is open to all members of the Ipswich community over the age of 12 years, the evaluation will target the adult population only as other cooking skills programs exist within the Greater Ipswich region that are specifically targeting children and adolescents in educational settings.

All participants will be required to consent prior to participation in the evaluation. A link to the questionnaire will be embedded within an email generated by the JMoF program database, inviting program participants to participate in the evaluation questionnaire.

Recruitment to both groups will be based on confirmed program start dates. As control participants are required to be on the program waitlist for 10 weeks and because the JMoF waitlist is sufficiently large to enable this to occur naturally, participants that are allocated a start date longer than 10 weeks ahead, will be automatically made a control, whilst persons commencing the program within 10 weeks will be assigned to the intervention group. Computer programmed “rules” in the JMoF participant database have been created to automate this process.

To show appreciation for participation, those intervention participants who complete the third and final questionnaire at six months post completion of the program will be sent a $20 “Good Guys” store charge card redeemable at any Good Guys store (white goods retailer) across Australia.

### Data collection

Data collection commenced in February 2012 following completion of piloting. Whilst the challenges of delivering online surveys are well documented [28], it was decided to be the most feasible and practical first method of delivery with subsequent delivery of a postal version to non-respondents and persons who do not have a working email address or access to a computer.

### Sample size

Given that the specific questions developed for this study to measure confidence to cook (cooking self-efficacy) have not been previously employed, a precise sample size calculation for this primary outcome could not be calculated given the absence of a priori baseline measures, measures of effect size and measures of variance (standard deviation).

Sample size calculations around the second primary outcome of a change in vegetable intake between the two groups assumed the use of a split-plot anova and F test for interaction given the wait list design. The literature does not provide clear guidance with regards to an expected effect size for a program of this nature [29,30]. However, as there is some evidence from Wrieden et al that an effect size of one serve per day may be too large for a program of this nature [20], sample size calculations are based on an effect size range likely to be achieved [29,30]. For an effect size of 0.5 serves a day, starting from a mean daily consumption at baseline of 2.5 serves per day [13,23], 250 subjects per group will be required at 80% power (0.05 significance). In the event that accrual is slower than expected, recruiting at least 140 participants will give 80% power for an effect size of 0.7 serves a day. There will be no analysis of the data before accrual has closed.

### Data analysis

Demographic and baseline characteristics will be summarised for both intervention and control groups using standard summary statistics (mean and standard deviation) and non-parametric statistics (medians and inter-quartile ranges) where applicable. Frequencies and percentages will be reported for categorical variables. The magnitude of change both within and between intervention and control groups for T1, T2 and T3 time-points will be assessed. For continuous data, such as fruit and vegetable intake, two sample t tests will be employed to compare means between intervention and control groups at each time point and paired t tests for within group comparisons. A split-plot in time Anova will be used as the basis for these t-tests. For categorical data, frequencies will be reported for intervention and control groups and chi squared analysis will provide between group comparison results. Regression analysis will be conducted to determine the potential contribution of specific demographic factors on the outcome variables of interest. All data analysis will be performed using STATA S.E. 12.0 statistical software.

### Qualitative study

### Research questions

To further understand how and why the JMoF program impacts on participants, the qualitative investigation will explore the following:

1. What are the expectations and experiences of participants?

2. What are the moderators, facilitators and barriers to behaviour change?

3. Were there any unanticipated outcomes?

### Study design

A longitudinal design will be employed for the qualitative study to follow program participants over the course of their JMoF journey. This will allow for prospective accounts of a participant’s experience and change over time [31,32]. Repeated semi-structured interviews will be conducted with participants. Up to three interviews will be conducted - prior to program commencement, on program completion and six months after completion.

### Participant interviews

The interview structure was generated to capture participant perspectives, to explore moderators, facilitators and barriers to behaviour change, and to capture any unanticipated outcomes from program involvement. As indicated in Table 1, the purpose of the sequential interviews is to understand participant expectations and experiences at different stages of program involvement. The interviews will be unstructured; however questions and prompts will be used to guide the discussion where appropriate. Table 1 lists the general discussion points used for each participant (interviews three and four will be based on previous discussions).

**Table 1:** Interview structure

**Interview timing**	**Discussion topics**
Interview 1: Prior to commencement of the program	Motivations for registering for the program, expectations of the program. Discussion of previous and current food and cooking experiences.
Contact: During program	Phone conversation to recruit participants to repeat interviews and to enquire how the course is going.
Interview 2: After program completed	Discussion around their program experience and whether program expectations were met. If participants experienced any changes in food and cooking behaviour and any unanticipated changes as a result of the program.
Interview 3: Six months after program completion	Discussion around whether any changes as a result of the program have been sustained in terms of food and cooking behaviour. Any unanticipated changes as a result of the program. Reflection on what was talked about in the last interview.

Interviews of approximately 30-40 minutes duration will take place in a public location that provides a comfortable environment for both interviewer and the participant. All interviews will be conducted by the same researcher and participants will be required to consent to both participation and recording of the interview. To show appreciation for participation in an interview, participants will be thanked with a $15 Coles supermarket gift voucher at each interview.

### Participant sampling

Purposive sampling will be employed initially to capture maximum variation [33] in factors considered likely to influence expectations and experiences of the JMoF program. These factors were captured in the participant questionnaire and included socio-economic status, age, gender, family structure, and cooking confidence level. Further sampling will be informed by themes emerging from the initial interviews and instructor observations about characteristics that seem to influence participant motivations and experience. Interview one will subsequently be conducted with approximately 10-15 participants. Participants who provide rich data in terms of insights and unique perspectives, and who are willing to continue, will be invited to progress to interviews two and three. In the event that there are insufficient numbers to progress (less than 6), new participants will be recruited from new enrolments.

### Participant recruitment

Participants who have completed the baseline quantitative study survey and have agreed to be contacted for an interview, will be eligible to participate as well as all participants who are within the first three weeks of commencing the program. The baseline survey data will assist in purposive selection for the qualitative interviews, using the criteria based on demographic and personal circumstances as previously described. Opportunistic purposive sampling will also be carried out during the researcher’s time in Ipswich during class observations. Selected participants will be contacted by phone or in person (in class context) and provided with information about the qualitative component of the study and invited to participate in the interviews. Written consent will be required prior to participation in the interview process.

### Data analysis

The interviews will be transcribed verbatim. The data will be managed with the assistance of qualitative software package NVivo 9 (NVivo 9 [program]: QSR International Pty Ltd 2011). Concurrent data collection and analysis will be conducted to allow for confirmation of emerging themes and clarification of any contradictory findings [34]. To contribute to the analytical process, the interviewer will record post-interview memos as reflections including contextual information, non-verbal factors of note, reflections on the interview process, and thoughts about emerging patterns or contradictions in the data [33,35].

The analysis of interview transcripts and interview memos will be conducted by the interviewer using inductive thematic analysis. The data will first be coded and then categorised to allow themes and patterns to emerge [34,36]. A second researcher will independently generate codes on a sub-sample of transcripts [37]. Comparisons will then be conducted and any differences will be discussed to achieve consensus in the final codes. The categorised data will then be reviewed to explore similarities and differences, to identify patterns and to determine whether there are specific relationships occurring between categories that together provide an overall conceptual picture of the impact of the JMOF program within the context of its unique setting and population. The resultant conceptual analysis will then be compared both to relevant theoretical frameworks and to the literature base to determine if it resonates with existing knowledge or makes a new contribution to the evidence.

### Integrated analysis

In addition to the separate analysis of the quantitative and qualitative results, integration of the respective findings will be conducted. This will involve examining consistencies and inconsistencies in the findings from each method [19,38] to build a more nuanced and comprehensive understanding of the JMoF program impacts and outcomes. This added depth and breadth will inform the conclusions drawn from the evaluation.

## Discussion

The evaluation of the JMoF program will contribute to the growing body of literature on the effectiveness of community-based cooking skills programs. It will employ a mixed methodology to draw on the strengths of both quantitative and qualitative study design to best capture and measure experiences, impacts and outcomes of cooking skills programs. The methods described herein will inform the research community about program outcomes and facilitate comparisons of results with other cooking skills programs conducted in comparable populations.

This study will also provide insights into practical considerations required when designing program evaluations in community settings. These include factors such as recruitment of a comparison group, minimisation of participant data collection burden, and the suitability and feasibility of selected data collection modes, which must be considered without compromising study design or program integrity.

There are both strengths and limitations to the evaluation design. Mixed methods studies as a paradigm can risk compromising methodological rigour if integration occurs at point of data collection and/or analysis and potentially undermines paradigm and process considerations [18,19]. This is not an issue in the current evaluation with integration only occurring in relation to sample identification and final integration of findings.

Whilst it is acknowledged that the use of a non-randomised quasi-experimental design makes the quantitative study vulnerable to sampling bias, practical limitations prevented the application of a randomised design. Despite this potential bias, the use of a waitlist control and pre and post measures support attribution of any changes to the program.

Potential selection bias associated with choice to participate or not in the quantitative study may also occur. However various methods were employed in an attempt to address this issue: providing participants with multiple options for survey completion, follow up of non-responders and the use of incentives.

In the quantitative study, there is no direct measure of cooking skills despite the JMoF program being a cooking skills program per se. However, there is currently no gold standard for the measurement of cooking skills in an adult population nor consensus on the definition of cooking skills or whether changes in it alone will predict the likelihood of changes in cooking behaviour [3]. Therefore confidence to cook which reflects self-efficacy, a relatively strong predictor of behaviour change, was the chosen measure for the evaluation as suggested by Winkler, Wrieden and Barton et al [3,20,21]. It is noted that even Barton et al’s confidence questions upon which some of the current survey questions are based, whilst considered reliable, have yet to be formally tested in the community setting [21]. Another limitation of the quantitative study is the reliance on self-reported measures. Yet lessons learnt from previous evaluations [20,21] suggest that the use of more intensive methods would likely overburden participants and lead to low participation rates.

In summary, the use, in this evaluation, of a mixed method, pre-post design with a waitlist control group will provide sufficient strength of evidence to assess the impact of the JMoF program on participants’ attitudes and behaviours. It will also make a contribution to the limited evidence base about the effectiveness of community-based cooking programs.

## Competing interests

The authors wish to declare that the evaluation has been commissioned by The Good Foundation.

## Authors’ contributions

All authors have contributed to project design and development, whilst AF and JH are responsible for project management and data collection. AF drafted the manuscript with JH and LG contributing to the qualitative sections. All listed authors reviewed the draft manuscript, then read and approved the final manuscript.

## Pre-publication history

The pre-publication history for this paper can be accessed here:

http://www.biomedcentral.com/1471-2458/13/411/prepub
